# Mechanisms of Action of Dorsal Root Ganglion Stimulation

**DOI:** 10.3390/ijms25073591

**Published:** 2024-03-22

**Authors:** Alaa Abd-Elsayed, Swarnima Vardhan, Abhinav Aggarwal, Madhurima Vardhan, Sudhir A. Diwan

**Affiliations:** 1 Department of Anesthesiology, School of Medicine and Public Health, University of Wisconsin, Madison, WI 53792, USA; 2 Department of Internal Medicine, Yale New Haven Health, Bridgeport Hospital, Bridgeport, CT 06605, USA; swarnima.vardhan@bpthosp.org (S.V.); abhinav.aggarwal@yale.edu (A.A.); 3 Advanced Spine on Park Avenue, New York, NY 10461, USA; sudhir.diwan63@gmail.com; 4 Argonne Leadership Computing Facility, Argonne National Laboratory, Lemont, IL 60439, USA; mvardhan@anl.gov

**Keywords:** chronic pain, neuropathic pain, dorsal root ganglion stimulation, neuromodulation

## Abstract

The dorsal root ganglion (DRG) serves as a pivotal site for managing chronic pain through dorsal root ganglion stimulation (DRG-S). In recent years, the DRG-S has emerged as an attractive modality in the armamentarium of neuromodulation therapy due to its accessibility and efficacy in alleviating chronic pain refractory to conventional treatments. Despite its therapeutic advantages, the precise mechanisms underlying DRG-S-induced analgesia remain elusive, attributed in part to the diverse sensory neuron population within the DRG and its modulation of both peripheral and central sensory processing pathways. Emerging evidence suggests that DRG-S may alleviate pain by several mechanisms, including the reduction of nociceptive signals at the T-junction of sensory neurons, modulation of pain gating pathways within the dorsal horn, and regulation of neuronal excitability within the DRG itself. However, elucidating the full extent of DRG-S mechanisms necessitates further exploration, particularly regarding its supraspinal effects and its interactions with cognitive and affective networks. Understanding these mechanisms is crucial for optimizing neurostimulation technologies and improving clinical outcomes of DRG-S for chronic pain management. This review provides a comprehensive overview of the DRG anatomy, mechanisms of action of the DRG-S, and its significance in neuromodulation therapy for chronic pain.

## 1. Introduction

Chronic pain, a pervasive neurological condition affecting around one-fifth of the global population, poses considerable challenges for patients, physicians, and society [1]. Despite a range of available treatments and emerging options, achieving sustained relief remains elusive for many [2]. Pharmacotherapy, behavioral and physical therapies, minimally invasive techniques, and even surgical interventions often fall short in providing adequate relief. The economic impact is substantial, with chronic pain accounting for over USD 600 billion in annual healthcare costs and remains a central factor in the continuing opioid crisis [3,4]. With a growing demand for enhanced therapeutic alternatives, neuromodulation advancements continue to evolve and transform significantly since the pioneering work by Shealy et al. [5]. These therapies aim to selectively target primary sensory neurons (PSNs) implicated in chronic pain and modify their biophysical characteristics, presenting a novel treatment avenue. Over the past decade, dorsal root ganglion stimulation (DRG-S) has surfaced as a pivotal advancement in the field of neuromodulation for chronic pain management [6].

The dorsal root ganglion (DRG), housing the cell bodies of the PSNs in the lateral epidural space of the spinal foramen, plays a crucial role in pain transmission to the CNS. Pathophysiologic changes in the DRG during chronic pain states alter the membrane properties and neurophysiological states of these PSNs [7]. Due to its fundamental role in sensory processing, particularly in nociceptive and neuropathic pain genesis, and its accessibility for clinical intervention, the DRG-S has emerged as an excellent target for pain management through neuromodulation [8,9,10]. In contrast to the conventional approach of applying the electrical field directly onto the dorsal columns of the spinal cord in tonic spinal cord stimulation (t-SCS), DRG-S requires the placement of a small electrode array in close proximity to the cell nuclei of afferent neurons within the intra-foraminal space along the dorsal aspect [11]. This targeted modulation of PSNs nestled within the DRG offers a promising, non-addictive, and reversible treatment option for cases of refractory pain unresponsive to standard medical interventions.

Addressing the gaps in comprehension of mechanisms and refining the clinical application of DRG-S could propel advancements in neuromodulation particularly for patients who do not achieve adequate pain relief through this technique, limiting their treatment options [9,10,12]. In this review, we offer an intricate exploration of the anatomy and mechanisms underlying DRG-S. These insights would help in understanding reasons for DRG-S inefficacy in certain patients, to enhance patient selection, and develop innovative DRG-S technologies that specifically target mechanisms to optimize pain relief universally, thereby contributing towards alleviating the substantial burden of chronic pain.

## 2. DRG Anatomy

**Figure 1:**
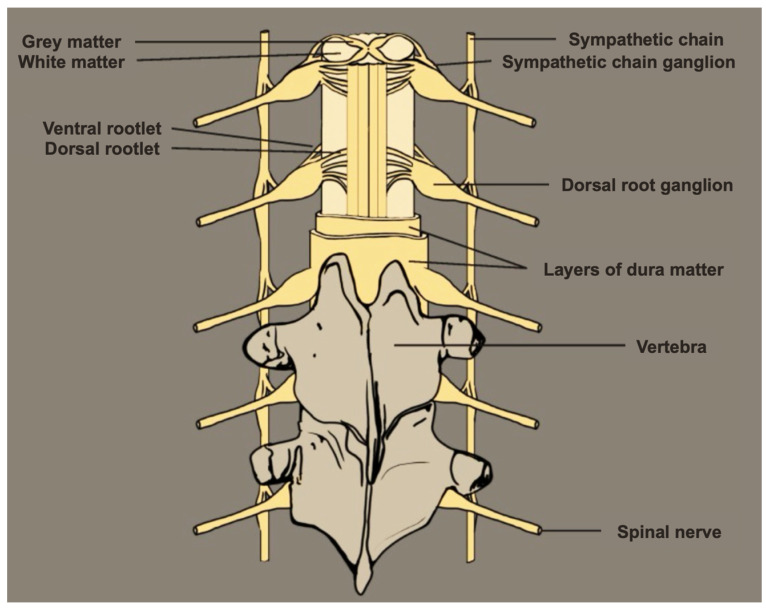
Anatomy of the dorsal root ganglion, vertebrae, grey and white matter. Figure reproduced from Ahimsadasan, N. et al., 2022 [16] under Creative Commons (CC BY) license.

The DRG is a crucial structure housing the cell bodies of highly compartmentalized PSNs, facilitating sensory transmission, including pain signaling [13]. Roughly the size of a small peanut, the DRG is enclosed within the dural sheath, nestled bilaterally at the intervertebral foramen along each of the 31 pairs of human spinal nerves. It is surrounded by a thin layer of cerebrospinal fluid (CSF) and situated within the fixed bony vertebral structures (neuroforamen), bridging the transition from the spinal cord and vertebral column to the peripheral nervous system (PNS) (Figure 1) [14,15,16].

**Figure 2:**
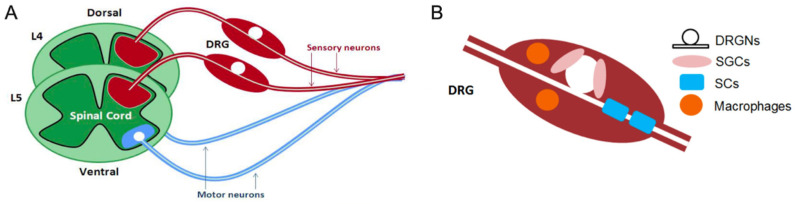
Schematic diagram delineating structure and cellular composition of dorsal root ganglion (DRG). (**A**) The DRG houses the cell bodies of the primary sensory neurons (PSNs), with central and peripheral axon extensions. (**B**) An illustration of the prominent cells within the DRG. The satellite glial cells (SGCs) surround the neuronal cell bodies, whilst the Schwann cells (SCs) ensheath and myelinate axon fibres, and finally the macrophages are present for the immune response. Image reproduced from Martin, S. et al. 2019 [20] under Creative commons [CC BY] license.

Besides their involvement in sensory neurotransmission, the DRG harbors non-neuronal cells and tissues crucial for supporting and preserving the functionality of the nucleus. Surrounding each neuronal cell body are satellite glial cells, providing support to the soma and establishing interconnected gap junctions implicated in sensitizing neighboring neurons [17,18]. Alongside the connective tissue that separates glial cells, a cluster of immune cells, primarily composed of macrophages and lymphocytes, along with blood vessels and bundles of sympathetic nerve fibres, constitutes the DRG (Figure 2) [19,20].

The distinctive attributes of the DRG, setting it apart from other targets along the sensory neural axis and rendering it more readily accessible for therapeutic approaches, encompass several factors. These include the peripheral localization of primary sensory afferents, the presence of a blood–nerve barrier that poses fewer limitations compared to the blood–brain barrier, and the presence of elongated axons housing distinct, targetable compartments [21].

### 2.1. DRG Neurons Are Pseudo-Unipolar

DRG neurons possess a distinctive pseudo-unipolar structure, characterized by a single axon (stem axon), which departs from the cell body and bifurcates at a unique T-junction, with one branch extending towards the peripheral target and the other traveling along the dorsal root into the central nervous system (CNS). This anatomic configuration facilitates the uninterrupted transmission of sensory information and is endowed with receptors for various neurotransmitters. The peripheral segment of the axon reaches receptor endings in the periphery, governing afferent signaling. Meanwhile, the central segment extends into the CNS, exhibiting extensive axonal arborizations within the spinal cord, culminating in synapses at ipsilateral or contralateral wide dynamic range neurons, inhibitory interneuron networks, and other dorsal horn targets [13].

Both the cell soma and the stem axon possess electrical excitability, hypothesized to prevent a conduction block at the T-junction. This excitability results in increased membrane capacitance and decreased membrane resistance, essential for spike propagation [22]. The T-junction of the DRG neuron serves multiple functions, potentially impeding electrical impulses from the peripheral nociceptor to the dorsal root entry zone of the spinal cord, participating in the transmission of electrical impulses, or acting as a low-pass filter for peripheral electrical information.

### 2.2. DRG Size

The dimensions of the DRG are intricately linked to the quantity of the neurons it encompasses, exhibiting variations contingent on its vertebral location [23]. In the cervical region, DRG size exhibits a gradient, demonstrating a progressive increase from a predominantly minimal presence, seen in over 70% at the C1 level, to reaching its maximum dimension at the C8 level [24]. Notably, the C8 DRG displays a greater volume (177 mm^3^) compared to the adjacent T1 DRG (144 mm^3^), nearly doubling the volume of the C5 DRG despite having a smaller receptive field [24]. Correspondingly, there is a progressive increase in the dimensions of the DRG from L1 to L5 levels in the lumbar region, ranging from approximately 4 mm × 4.5 mm at L1 to 5.5 mm × 10 mm at L5. Conversely, the sacral DRG exhibits a reduction in size from S1 to S4, with the S1 DRG measuring around 6.5 mm × 13 mm in contrast to 5 mm × 3 mm at S4 [25].

### 2.3. DRG Soma Size

DRG neurons also exhibit varied soma sizes, spanning approximately from 20 to 100 μm, and are typically categorized as small (<25 μm), medium-sized (25–35 μm), or large (>35 μm) [13]. This variation in sizing directly correlates with the degree of axon myelination, thereby influencing the conduction velocity. Aβ, Aδ, and C fibres relay the peripheral sensation information towards their corresponding somas within the DRG. The largest-diameter, myelinated Aβ primary afferents possess a low mechanical threshold and transmit non-painful tactile and proprioceptive information to the spinal cord [26]. Myelinated Aδ fibres notably demonstrate higher conduction velocities, facilitating the transmission of acute non-noxious information, such as responses to temperature, mechanical stimuli, and chemical triggers, to the DRG. Pain-inducing stimuli are primarily conveyed by unmyelinated C fibres and a subset of myelinated Aδ fibres. The unmyelinated C fibres, characterized by smaller diameters and slower conduction velocities, predominantly play a role in relaying more diffuse and deeper secondary pain signals that manifest after an injury [22].

### 2.4. DRG Position in the Foramina 

The DRG typically maintains a consistent location, primarily situated in the superior part of the foramen in close proximity to the rostral pedicle [27]. In the thoracic and lumbar levels, the DRG resides in close proximity to the vertebral pedicle. For instance, in approximately 75% of cases, the L5 DRG is intraforaminal, with only 6% situated extraforaminal [28]. However, sacral DRGs exhibit distinct categorizations—described either as intracanalar (positioned medial to the medial border of the sacral pedicle) or intraforaminal (situated lateral to the medial border of the sacral pedicle). The S1 DRG is intraforaminal in approximately 60% of instances, while at the S2 level, the majority are within the spinal canal (ranging from 50% to 85%). At the S3 and S4 levels, all DRGs are contained within the canal [27,29].

### 2.5. DRG Vasculature

The DRG receives its blood supply from the spinal branch of the dorsal trunk of segmental arteries. Blood circulation is facilitated by the radiculo-medullary branches that supply the spinal nerve, traversing along the ventral aspect of the DRG and nerve roots [30]. Upon penetrating the DRG, these vessels form a subcapsular capillary plexus before delving deeper and branching into intra-ganglionic vessels, establishing an intricate capillary network that intimately engagesing with sensory neurons [31].

The DRG houses fenestrated capillaries and lies beyond the blood–brain barrier, facilitating direct access of molecules from the vascular system to the DRG. This structural setup ensures an adequate blood supply essential for the synthesis and transport of proteins [32]. It has been noted that the regulation of blood flow to the DRG is orchestrated by muscular sphincters, dynamically adjusting flow rates in accordance with functional and metabolic requirements. This intricate vascular structure of the DRG plays a vital role in providing metabolic intermediates necessary to sustain the relatively high metabolic requirements of the cellular constituents of the ganglion [33].

### 2.6. DRG Stimulation (DRG-S) versus Spinal Cord Stimulation (SCS)

Over the past decade, extensive research has spotlighted DRG as a promising focal point for neuroelectrical modulation with the first patent for a pioneering device designed to stimulating the DRG filed in 2004 [34,35,36]. The application of a lead onto the DRG has necessitated significant alterations from the conventional SCS systems, such as a reduced lead diameter, smaller contact size, enhanced flexibility, and the development of a novel implantation technique. Notably, the cellular mechanism underlying DRG-S diverges from the reliance on gamma-aminobutyric acid (GABA) release within the dorsal horn of the spinal cord, a characteristic feature of conventional SCS, with reduced levels of this inhibitory neurotransmitter noted following SCS [37,38].

DRG-S demonstrates several distinct advantages over SCS. Postural adjustments can affect the proximity between electrodes and the dorsal column, influencing the scope of neural tissue stimulation in traditional SCS. Configurations in SCS that diminish this distance expand the neural volume stimulation, recruiting higher-threshold fibres, and resulting in excessive perceived stimulation, resulting in side effects, such as, unintended paresthesias, along with muscular cramping [39,40]. Conversely, DRG-S demonstrates limited susceptibility to postural variations. The placement of DRG leads within the neuroforamen, accompanied by minimal CSF space and strengthened by surrounding vertebral structures, mitigates the concerns regarding lead displacement caused by bodily movements [41].

Physiologically, in the spinal cord compartment, the elements with the highest to lowest conductivity of electricity are the CSF, longitudinal fibres, and grey matter. The efficiency of DRG-S stems from a diminished current loss across thinner subdural CSF layers, as the proximity of leads to the neural target decreases the amplitude requirement compared to SCS. The lower power demand of DRG-S, up to 92.5% less than SCS, can potentially extend battery life, reduce healthcare costs, and enhance treatment adherence. Given the potential patient burden and impact on treatment outcomes, these considerations underscore the significance of DRG-S in pain management. These considerations are crucial given that frequent engagements with the healthcare system create a significant burden on the patients and exacerbate their contemplations regarding pain, potentially leading to inferior treatment outcomes [42,43].

The understanding of stimulation parameters, particularly stimulation frequency, in DRG-S, is still evolving due to the novelty of the technique and the peculiarity of the target structure. Various frequencies have been investigated in DRG-S, with a randomized double-blind clinical trial indicating that 20 Hz stimulation was more effective than frequencies of 40 Hz, 60 Hz, and 80 Hz [44]. It has been hypothesized that stimulation at even lower frequencies (≤5 Hz) may activate action potentials (APs) across all types of low-threshold mechanoreceptor (LTMR) fibres, potentially eliciting a broader engagement of the inherent physiological inhibitory systems of the body via opioid receptor activation. This proposed mechanism distinguishes itself from traditional SCS, which primarily targets Aβ fibres within the dorsal column [45]. In fact, a recent case series by Chapman et al. suggested potential therapeutic advantages of employing even lower frequencies, such as 4 Hz, for pain relief, prompting further investigation [46]. However, a subsequent randomized double-blind clinical trial failed to show significant differences between 4 Hz and the conventional 20 Hz stimulations in terms of pain relief. The authors concluded that the study may have been underpowered to detect subtle differences, suggesting that further studies with extended observation periods may be warranted to comprehensively assess the nuances of frequency response in DRG-S therapy [47].

DRG-S arises as a prospective intervention for individuals who have encountered limited enduring advantages from SCS. The effectiveness of DRG-S as a salvage therapy might be explained by a distinctive, targeted mechanism of action, which focuses on a distinct anatomical structure compared to SCS [48].

### 2.7. Clinical Applications of DRG-S

The majority of the research on the application of DRG-S has focused on its effectiveness in managing complex regional pain syndrome (CRPS). CRPS is typified by pain that is disproportionate to the initiating event and is often accompanied by abnormal sensory responses such as allodynia, along with vasomotor, sudomotor, and/or motor/trophic symptoms. The seminal ACCURATE Trial conducted by Deer et al. demonstrated the superiority of DRG-S over traditional dorsal column SCS in managing focal pain attributed to CRPS I or II in the lower extremities, as evidenced by outcomes observed over a 12-month follow-up period [9]. In 2016, the US Food and Drug Administration (FDA) granted approval for the use of DRG-S in treating lower extremity pain associated with CRPS [49].

Recent studies have underscored the favorable outcomes of DRG-S neuromodulation compared to SCS across various conditions, including chronic pelvic pain, painful diabetic neuropathy, restless leg syndrome, and post-surgical or traumatic painful scars. These studies have demonstrated notable enhancements in quality of life, increased patient satisfaction, reduced medication dependency, and long-term cost-effectiveness [50,51,52,53]. Failed back surgery syndrome (FBSS) is usually refractory to conservative medical management. There are several studies which show growing evidence for neuromodulation in the treatment of FBSS and non-surgical low back pain (NSLBP), demonstrating improvement in pain scores, functional and psychological measures, and overall quality of life. While SCS continues to be the preferred therapy in FBSS and NSLBP, recent prospective studies highlight the potential role of DRG-S as a secondary treatment option, particularly following unsuccessful SCS interventions [54,55].

However, the existing evidence largely stems from case reports and observational studies involving small sample sizes and short follow-up durations. There is a pressing need for higher-quality research, such as meta-analyses and randomized controlled trials (RCTs), to establish stronger recommendations regarding the use of DRG-S in the management of chronic pain unrelated to CRPS.

## 3. DRG Mechanisms

DRG-S represents an important neuromodulatory intervention within the pain management armamentarium for addressing chronic and refractory conditions. Emerging insights underscore the nuanced nature of neuromodulatory therapies, indicating that DRG-S, like its counterparts, operates through a complex interplay of mechanisms rather than a singular pathway. Accumulating evidence suggests that DRG-S potentially engenders analgesic effects by enhancing the attenuation of painful signals at the T-junction of nociceptive neurons through low-pass filtering, stimulating the post-synaptic activation of pain-gating circuitry in the dorsal horn and conceivably within the DRG itself, and by modulating the intrinsic excitability of DRG neurons.

### 3.1. Effects at T-Junction

**Figure 3:**
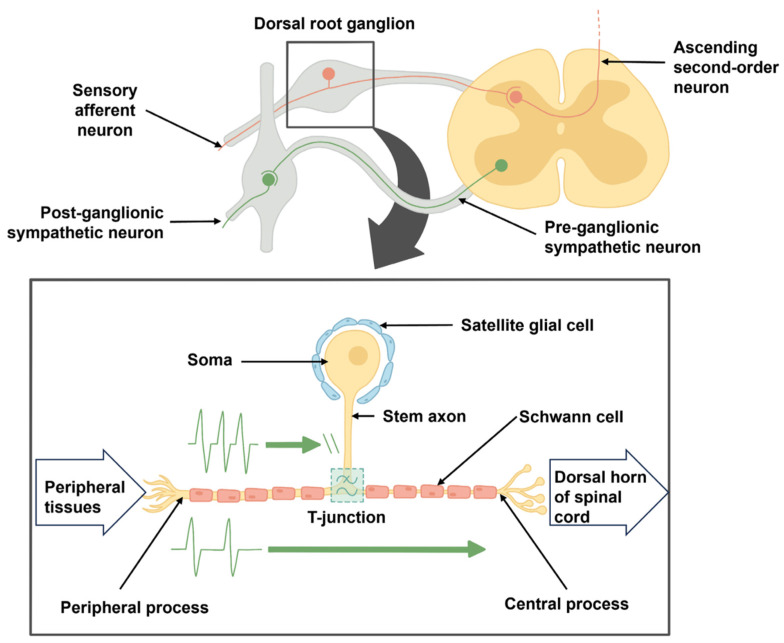
The figure illustrates the location of DRG in the spinal cord. DRG neuron has a unique pseudo-unipolar structure characterized by a single axon which bifurcates at the T-junction. The T-junction serves as a site of selective modulation of electrical impulses originating from the periphery transmitted via the axon. Figure reproduced from Sørenstua, M. et al., 2023 [58] under Creative Commons (CC BY) license.

The T-junction serves as a large node of Ranvier, where the peripherally projecting axon typically exhibiting a larger diameter compared to the spinally projecting axon, facilitates the selective modulation of electrical impulses originating from the periphery, enabling their potential bypass, blockage, or filtration by the soma (Figure 3) [22,56,57,58].

In vivo intracellular recordings have elucidated the existence of a low-pass filter positioned at this junction, whose adaptability becomes more permissive in contexts of chronic pain [59]. The local expression of ion channels housed in the cell bodies of the DRG neurons influences C-fibre signaling and facilitates the invasion of peripherally generated action potentials into the soma, thereby regulating the transmission of afferent signals from the periphery to the central axon [60]. Considerable evidence from myelinated and unmyelinated sensory neurons suggests that the T-junction serves as a site where the safety margin for spike propagation is diminished and may contribute to the low-pass filtering of action potentials. In myelinated afferents, for example, orthodromic propagation of action potentials can trigger supplementary spikes within the initial segment. These additional spikes may then reverberate towards the T-junction, potentially obstructing subsequent orthodromic action potentials. This phenomenon of self-induced occlusion seems to enhance both short and long inter-spike intervals, while simultaneously diminishing the number of intermediate inter-spike intervals [59].

In unmyelinated afferents, an amalgamation of morphological and electrophysiological attributes, encompassing factors like length of the stem axon and the presence of slow hyperpolarizing conductances, contributes to the creation of a low-pass filtering effect on orthodromically propagating action potentials [61]. DRG-S is postulated to offer analgesic benefits by potentially intensifying this filtering characteristic within the DRG neurons implicated in pain pathophysiology, thereby further modulating pain perception. For instance, in conditions such as CRPS, there is a reduction in filtering across the T-junction of the DRG coupled with increased hyperexcitability.

Endogenous opioids such as endorphins and enkephalins play a crucial role in modulating nociceptive transmission and pain perception by binding to μ, γ, and κ opioid receptors distributed across presynaptic terminals and interneurons within the dorsal horn. The enkephalins activate Aβ neurons, whereas the Aδ and C LTMRs trigger dynorphin release to suppress nociceptive signaling. μ opioid receptors, found presynaptically on select Aδ and C fibres, are responsive to both endorphins and enkephalins. Aβ neurons are linked to δ opioid receptors, while κ opioid receptors are activated by Aδ and C receptors [62,63]. It is proposed that the efficacy of DRG-S in CRPS is due to the inhibition of noxious and autonomic afferent signals at the T-junction and linked to the activation of the endogenous opioid systems in the dorsal horn. In CRPS, it has been observed that repetitive electrical stimulation of afferent C fibres transiently increases the excitability of wide-dynamic-range neurons, a phenomenon known as “windup.” This phenomenon signifies a type of central sensitization and plays a role in chronic pain development. The decrease in windup following DRG-S exerts an inhibitory effect on neuronal sensitization to repetitive noxious stimuli [64].

The sequential amplification of T-junction filtering encompasses the orchestrated interplay of calcium and potassium channels. Stimulation-triggered action potentials originating within the soma trigger a cascade of events, initiating calcium influx. This heightened calcium concentration significantly augments the conductivity of small conductance calcium-activated potassium (K_SK_) channels, thereby inducing sustained somatic hyperpolarization that distinctly influences the dynamics of both the stem axon and the T-junction, and contribute to filtering at the T-junction [65]. This, in turn, diminishes the input resistance of the soma and the T-junction within nociceptive PSNs, thereby limiting the propagation of high-frequency pain signals towards the central nervous system [59,66].

An alternative hypothesis revolves around the concept of filtration occurring specifically at the T-junction of C neurons. This hypothesis postulates two potential mechanisms: either DRG-S directly intervenes in modulating or disrupting the ongoing activity within C neurons, or DRG-S indirectly influences C-neuron activity through an alternate mechanism. Notably, trains of action potentials exhibit a higher frequency of propagation in the orthodromic direction when traversing through the T-junction compared to the antidromic direction, attributed to the reduction in axonal diameter from the peripheral to the spinal axon [59,65].

There also exists a minimum voltage threshold, termed the excitation threshold, that governs the propagation of an action potential along an axon. Any membrane potential exceeding this threshold constitutes the “safety factor” [59]. Notably, the safety factor is reduced at branching points like the T-junction, elevating the likelihood of interruptions in action potential propagation and neural conduction. It has been established that both myelinated and unmyelinated neurons of DRG cells possess larger diameter peripheral processes with lower impedance, contrasted by a smaller-diameter central processes exhibiting higher impedance. This disparity in impedance contributes to the neural modulation at the T-junction of DRG neurons [67]. Here, electrical signals transitioning from peripheral (low-impedance) to central (high-impedance) axonal fibres results in the attenuation of high-frequency action potentials [61]. Overall, these findings suggest that branch points such as the T-junction within DRG neurons act as a protective switch or a low-pass filter for high-frequency action potentials encoding noxious stimuli, thereby shielding the soma from excessive input, and providing a defensive mechanism to attenuate the perception of pain [68].

### 3.2. Effect on Nociceptive Neurons: Suppressing PSN Hyperexcitability

The aftermath of nerve damage encompasses a spectrum of changes within both the nociceptors and the central nervous system. Among these alterations, a well-documented phenomenon involves the hyperexcitability observed within DRG neurons subsequent to injuries affecting peripheral axons of the DRG. There is a proposition suggesting that this augmented excitability of DRG neurons post-injury might play a contributory role in the manifestation of neuropathic pain [22]. Moreover, it has been theorized that the increased expression of sodium channel Nav1.3 contributes to the abnormal hyperexcitability observed in injured DRG neurons [69].

An additional proposed mechanism suggests that DRG-S elicits analgesia by diminishing ectopic activity within nociceptive neurons. While certain PSNs exhibit spontaneous activity in a healthy state, nerve injury triggers a significant surge in ectopic activity among nociceptive neurons within the DRG [70,71]. In unmyelinated C-type neurons, this activity manifests as subthreshold membrane potential oscillations (SMPOs) and irregular or bursting action potentials [60]. This aberrant surge in activity potentially originates from the inflammatory cascade triggered by nerve damage, precipitating the release of nerve growth factor and the consequent upregulation of late and persistent sodium channels within nociceptive PSNs [72,73,74]. It is believed that this ectopic activity originates in the soma, potentially involving nearby axons or the T-junction, with ensuing action potentials propagating towards the central nervous system, contributing to sensations of neuropathic pain [75,76]. DRG-S, known to dampen the sensory neuron excitability, might effectively suppress this aberrant ectopic activity, thereby offering analgesic relief. Intriguingly, this mode of DRG-S operates in a seemingly paradoxical manner, where direct stimulation of the stem and soma generates excitation which results in a hyperpolarization effect that effectively impedes pain signals originating from the periphery and attenuates the ectopic activity originating from the soma.

Another hypothesized mechanism underlying allodynia revolves around the development of inherent spontaneous activity observed within Aβ-neurons, traditionally characterized by their non-transmission of pain signals. Consequently, this clinical expression of neuropathic pain results in the perception of pain from stimuli that were previously non-painful [77,78]. Contemporary investigations indicate the potential involvement of the DRG in the genesis of neuropathic pain, marked by heightened excitability and the occurrence of spontaneous ectopic firing within PSNs [65].

### 3.3. Effect on Glial Cells

The glial cells within the PNS primarily consist of Schwann cells and satellite glial cells (SGCs). Research focused on PNS injuries has revealed significant contributions to the development and persistence of pain from resident macrophages and inflammatory cells that infiltrate this neural network [79]. The DRG encases neurons with distinct membrane characteristics, encircled by SGCs, typically acting as sentinels safeguarding these neural entities. However, post-injury, these glial cells possess the capacity to mobilize immune response cells, thereby modulating the inflammatory milieu within the DRG [80]. Additionally, studies denote that injury to peripheral nerves triggers the release of neurotrophic factors from these glial cells. These factors elicit a proliferative response in the glia, leading to the mechanical compression of sensory neuron cell bodies. This compression augments the nerve irritation within the DRG, perpetuating a sustained influx of immune cells and inflammatory cascades. These factors collectively establish a cyclical pattern of inflammation within the DRG, persisting even upon complete resolution of the peripheral nerve injury [13].

Despite their physical proximity, the cell bodies of DRG neurons typically operate in isolation due to the encasing layers of SGCs. Yet, a small subset (constituting 4–9% depending on the species) of DRG neurons shares a common glial sheath, forming clusters with one or two other neurons. This arrangement opens avenues for the potential “cross-excitation” or “cross-depolarization” of multiple DRG neurons by a single stimulus [35,81]. Studies have shown that almost all DRG neurons experience subthreshold excitation coinciding with the activation of the neighboring cell bodies. Up to 90% of DRG neurons depolarize when a stimulus is applied to the axon of a neighboring neuron within the same ganglion [35,82]. Notably, the gap between SGCs and the neuronal surface is approximately 20 nm, akin to the distance observed in synaptic spaces. This close configuration allows the SGCs to actively engage in cellular communication within the DRG. Previously considered primarily as support for maintaining the cellular equilibrium of DRG neuron cell bodies, recent studies have revealed the active and influential roles played by SGCs in the normal physiological functioning of these ganglia. Consequently, due to this dynamic interaction, dysfunction in SGCs significantly impacts the DRG neuron activity, this contributing to the initiation and perpetuation of neuropathic pain [81].

Nerve damage or inflammation triggers the activation of SGCs within the sensory ganglia. In response to injury, SGCs upregulate the expression of the astrocyte marker glial fibrillary acidic protein (GFAP), signaling increased glial reactivity and the coupling of SGCs through gap junctions [83]. This augmented intercellular connectivity among SGCs contributes to a reduction in the pain threshold [84]. Additionally, activated SGCs also release proinflammatory cytokines, including IL-1β, IL-6, TNF, and fractalkine, eliciting augmented neuronal firing rates and excitability, while also simultaneously coordinating the recruitment of circulating immune cells such as T cells, neutrophils, and macrophages [85,86,87,88].

### 3.4. Orthodromic and Antidromic Effects

A crucial property of nerve conduction involves the activation of primary afferent nerve fibres distant from the terminal DRG, resulting in the generation of bidirectionally conveyed action potentials. This includes orthodromic input towards the spinal cord and antidromic conduction in the peripheral axons. These signals elicit a calcium-dependent release of neurotransmitters. Additionally, orthodromic activity within the intraspinal circuit in the dorsal horn activates GABAergic neurons, which receive input from nociceptive Aδ and C afferent fibres. This suggests that the afferent drive is subject to modulation by the GABAergic neurons [89,90,91].

Action potentials originate in the peripheral terminals of the DRG neurons, with certain small afferent sympathetic fibres capable of conducting action potentials retrogradely along the same axon, a phenomenon termed as the dorsal root reflex (DRR). Gotch and Horsley first described this centrifugally conducted activity in dorsal roots in 1931 [92,93]. Dorsal root reflexes (DRRs) are thought to play an important role in neurogenic inflammation. Neurogenic inflammation is triggered by substances released from sensory nerve terminals and is characterized mainly by arteriolar vasodilation and plasma extravasation. Notably, neuropeptides, such as substance P and calcitonin gene-related peptide (CGRP) are potent vasodilators which are present in the peripheral terminals of nociceptive fibres and are released in response to antidromic stimulation. This process is implicated in facilitating the effects of the antidromic propagation of action potentials with DRG-S [94,95].

### 3.5. Effects on GABA

The effectiveness of DRG-S may be attributed to the release of GABA within the DRG, the expression of GABA-A receptors in sensory neurons, and the subsequent blocking of action potential propagation through the activation of GABA-A receptors within the DRG [96,97,98,99,100,101]. The Gate Control Theory highlights the integral role of inhibitory GABA-ergic interneurons in modulating nociceptive afferents, serving as a fundamental regulatory element in the SCS of dorsal columns. Stimulation of the ascending branch of non-nociceptive large Aβ fibres in the dorsal column induces antidromic activation, initiating synaptic interactions with GABA-ergic interneurons [97,98,99,100,101]. Activation of GABA-ergic inhibitory interneurons in the superficial laminae of the dorsal horn by Aβ fibres amplifies GABA release, leading to elevated extracellular GABA levels [100]. Importantly, peripheral nerve injury disrupts the innate GABA-ergic inhibition, culminating in heightened neuronal excitability within the spinal dorsal horn—a pivotal factor contributing to neuropathic pain [102,103,104,105].

Koetsier et al. conducted a pioneering experimental study assessing the intracellular GABA levels within the spinal dorsal horn during the peak period of pain relief induced by DRG-S in a neuropathic pain model of painful diabetic polyneuropathy (PDPN). Their investigation utilized immunohistochemical analysis to examine the impact of DRG-S on the intensity of intracellular GABA-staining levels specifically within laminae 1–3 of the L4-L6 spinal levels in animals with PDPN. The study results revealed no statistically significant variance in GABA-immunoreactivity between DRG-S and Sham-DRG-S, neither on the ipsilateral nor contralateral sides. Based on these findings, the study concluded that DRG-S does not elicit GABA release from spinal dorsal horn cells. This observation suggests that the mechanisms facilitating pain relief through DRG-S differ from those associated with conventional SCS [37].

### 3.6. Supraspinal Effects

DRG-S potentially exerts an influence on supraspinal structures, as evidenced in both animal and human studies [106]. The impact of DRG-S extends to the ascending lateral pathways, pain matrix regions, and descending modulatory pathways, as demonstrated through diverse measurement techniques, including LEP, fMRI, and 18 FDG PET/CT. This observation suggests a convergence in supraspinal mechanisms shared between both SCS and DRG-S [107,108]. The prevailing hypothesis concerning the supraspinal mechanism of action of SCS frequently revolves around the modulation of descending nociceptive inhibitory pathways, followed by adjustments in the ascending medial and lateral pathways [109].

In the first study exploring the supraspinal involvement in DRG-S, Pawela and colleagues conducted functional magnetic resonance imaging (fMRI) within a rat model of DRG-S. Their study aimed to evaluate the impact of DRG-S on the blood oxygen-level dependent (BOLD) signal elicited by the noxious electrical stimulation of the hindlimb. This research marked the initial identification of specific brain regions (including the primary/secondary somatosensory cortex, retrosplenial granular cortex, and basal ganglia) responsive to neuromodulation at the DRG level. Furthermore, the findings suggested potential mechanisms for targeted DRG electrical stimulation or ganglionic field stimulation as a therapeutic approach for chronic pain management [106]. Morgalla et al. conducted a prospective study involving 12 patients experiencing unilateral localized neuropathic pain in the lower limbs or inguinal region. Their investigation highlighted that DRG-S contributed to the restoration of normal laser-evoked potential (LEP) physiology by mitigating the hyperactivity of DRG neurons, consequently diminishing the influence of diffuse noxious inhibitory control on the second-order neurons [108].

Verills et al. conducted a retrospective study involving 39 patients to determine if the DRG-S is paresthesia independent. Their findings revealed that 87% of patients reported the absence of paresthesias during follow-up periods at three-, six-, and twelve-month intervals after treatment. Notably, the study observed an average pain relief of 63.1% after 12 months. One plausible mechanism underlying these findings suggests that DRG-S, which does not elicit paresthesias, potentially attenuates the processing of sensory information at supraspinal levels. This observed effect appears to complement the modulation of painful afferents specifically at the DRG level [110].

### 3.7. Effects on Ion Channels

The key channels involved in the transmission of action potentials within DRG neurons encompass sodium, potassium, and calcium channels. In response to nerve injury, the amplification of ion channel expression induces neuronal hyperexcitability and sustained neuronal stimulation within the DRG. This enhanced sensitivity crucially contributes to the persistent perception of chronic pain [111,112]. Multiple types of sodium channels are expressed in DRG neurons which are involved in spontaneous action potential generation and hyperexcitability within damaged sensory axons. For instance, there is increased expression of tetrodotoxin-insensitive sodium channels, such as Nav1.8, which significantly contributes to the emergence of ectopic spontaneous activity and neuropathic pain [113]. The voltage-gated calcium channels and glutamate receptors, located on the presynaptic membranes, regulate neurotransmitter release which plays a crucial role in modulating synaptic transmission. Following an injury to peripheral afferent fibres, in vitro studies indicated that DRG neurons demonstrate hyperexcitability and manifest ectopic firing patterns [114]. Neuropathic pain is intricately linked with increased excitatory (glutamatergic) transmission. The glutamate release by C fibres elicits an amplified response in dorsal horn neurons known as the “windup phenomenon” [115]. Pain alleviation during DRG-S might be attributed to the decrease in neural excitability and the ectopic firing rate within the neuropathic DRG [72,116,117].

### 3.8. Reversal of Cytokine Release

Injury to peripheral afferent fibres triggers the activation of microglia within the DRG. Subsequently, this activation initiates a cascade of cytokines, resulting in inflammation and the onset of neuropathic pain. Targeted electrical stimulation of the DRG counteracts the microglial activation within this region, consequently interrupting the aberrant cytokine cascade responsible for driving the genesis of neuropathic pain. Amir and colleagues have demonstrated that damage to peripheral afferent fibres induces irregular neural patterns marked by oscillatory and burst activity within the DRG [72]. It is theorized that the electrical stimulation of the DRG, akin to the effects seen in deep brain stimulation (DBS), could modulate this aberrant electrical activity by stabilizing the microglia and preventing the release of cytokines, potentially alleviating chronic pain [118]. Additionally, a separate study demonstrated that external electrical stimulation of the DRG regulates both tonic and burst activities in pseudo-unipolar neurons within the DRG [7]. An in-vitro animal study showed that the analgesic effect of DRG-S could be attributed to its ability to modulate nociceptive signals through the regulation of calcium influx locally. This modulation results in the attenuation of both the firing frequency and amplitude of multiple action potentials within the DRG neurons, leading to decreased neuron excitability and slowed nerve conduction velocity compared to the baseline levels prior to stimulation [66].

## 4. Conclusions

DRG-S is emerging as a promising and innovative neuromodulation technique in the arsenal of pain physicians for addressing intractable chronic pain. Like other neuromodulation therapies, DRG-S operates through multiple mechanisms, as evidenced by a growing body of literature. This review aimed to synthesize the latest evidence on the proposed mechanisms of DRG-S, identify gaps in understanding, and highlight unexplored avenues for future investigation. While DRG-S offers several potential advantages over conventional SCS systems, including direct anatomical targeting and reduced risk of complications, its current approval is limited to managing pain in CRPS. However, its application in various other chronic pain conditions is evolving. Despite its promise, the quantity of evidence-based literature delineating its indications, mechanisms, and advantages remains limited. Therefore, comprehensive mechanistic and evidence-based clinical research is urgently needed to bridge our significant knowledge gaps and expand the therapeutic potential of DRG-S. Embracing this emerging paradigm of therapeutic techniques exploiting DRG-S will undoubtedly enrich our armamentarium for chronic pain management, reinforcing the imperative for ongoing research and development in this ever-advancing field.

## Abbreviations

AP	Action potentials
BOLD	Blood oxygen-level dependent
CGRP	Calcitonin gene-related peptide
CNS	Central nervous system
CRPS	Complex regional pain syndrome
CSF	Cerebrospinal fluid
DBS	Deep brain stimulation
DRG	Dorsal root ganglion
DRG-S	Dorsal root ganglion stimulation
DRR	Dorsal root reflex
FBSS	Failed back surgery syndrome
FDA	Food and Drug Administration
fMRI	Functional magnetic resonance imaging
GABA	Gamma-aminobutyric acid
GFAP	Glial fibrillary acidic protein
LEP	Laser-evoked potential
LTMR	Low-threshold mechanoreceptor
NSLBP	Non-surgical low back pain
PDPN	Painful diabetic polyneuropathy
PNS	Peripheral nervous system
PSN	Primary sensory neurons
RCT	Randomized controlled trial
SCS	Spinal cord stimulation
SGC	Satellite glial cells
SMPO	Subthreshold membrane potential oscillations
